# Eight things you should never do in a monitoring program: an Australian perspective

**DOI:** 10.1007/s10661-022-10348-6

**Published:** 2022-08-22

**Authors:** David B. Lindenmayer, John Woinarski, Sarah Legge, Martine Maron, Stephen T. Garnett, Tyrone Lavery, Jaana Dielenberg, Brendan A. Wintle

**Affiliations:** 1 Fenner School of Environment & Society, The Australian National University, Australian Capital Territory, Canberra, Australia; 2 Research Institute of Environment and Livelihoods, Charles Darwin University, Northern Territory, Australia; 3 School of Earth and Environmental Sciences, The University of Queensland, St Lucia, Australia; 4 Centre for Biodiversity and Conservation Science, The University of Queensland, St Lucia, QLD Australia; 5 School of Ecosystem and Forest Science, University of Melbourne, Parkville, VIC Australia

**Keywords:** Long-term studies, Ecological management, Threatened species conservation, Environmental return on investment, Data integrity, Australia

## Abstract

Monitoring is critical to gauge the effect of environmental management interventions as well as to measure the effects of human disturbances such as climate change. Recognition of the critical need for monitoring means that, at irregular intervals, recommendations are made for new government-instigated programs or to revamp existing ones. Using insights from past well-intentioned (but sadly also often failed) attempts to establish and maintain government-instigated monitoring programs in Australia, we outline eight things that should never be done in environmental monitoring programs (if they aim to be useful). These are the following: (1) Never commence a new environmental management initiative without also committing to a monitoring program. (2) Never start a monitoring program without clear questions. (3) Never implement a monitoring program without first doing a proper experimental design. (4) Never ignore the importance of matching the purpose and objectives of a monitoring program to the design of that program. (5) Never change the way you monitor something without ensuring new methods can be calibrated with the old ones. (6) Never try to monitor everything. (7) Never collect data without planning to curate and report on it. (8) If possible, avoid starting a monitoring program without the necessary resources secured. To balance our “nevers”, we provide a checklist of actions that will increase the chances a monitoring program will actually measure the effectiveness of environmental management. Scientists and resource management practitioners need to be part of a stronger narrative for, and key participants in, well-designed, implemented, and maintained government-led monitoring programs. We argue that monitoring programs should be mandated in threatened species conservation programs and all new environmental management initiatives.

## Introduction

Good monitoring is fundamental to sound ecological management, threatened species recovery, and environmental reporting (Gardner, 2010; Legge et al., 2018). Monitoring is especially important in a world that is changing with increasing rapidity, particularly in response to climate change and increasing human use of natural resources (Bergstrom et al., 2021; Intergovernmental Science-policy Platform on Biodiversity and Ecosystem Services (IPBES), 2019). There are five key reasons to monitor: knowing the state of a system; understanding the way a system works (including the effectiveness of management interventions); raising awareness about an issue; engaging the public and leveraging effort; and discovering threats or opportunities (Possingham et al., 2012). Monitoring is deemed as a critical part of global initiatives such as the Convention on Biological Diversity (Moreaux et al., 2018), national-level directives on the management of biodiversity (e.g. Federal Office for the Environment, 2017), the maintenance of the integrity of particular ecosystems (e.g. Phalan et al., 2019), and the protection of particular endangered species (Lavery et al., 2021; Scheele et al., 2019).

Unfortunately, poorly designed and poorly implemented monitoring programs have characterized many large-scale environmental management initiatives around the world, including agri-environmental schemes (Whitfield, 2006), river restoration programs (Bernhardt et al., 2005), and landscape remediation in the face of secondary salinity (Ghassemi et al., 1995; Pannell & Roberts, 2010). Sometimes the purpose of a monitoring program has not been clearly articulated, increasing the chances that the monitoring is a waste of time and money (McDonald-Madden et al., 2010; Wintle, 2018). Moreover, monitoring programs have often been starved of resources, being the last initiative funded in natural resource management programs and the first item cut when budget pressures arise (Lindenmayer & Likens, 2018).

The problems of poor-quality environmental monitoring at a global level have been repeated at continental, national, and smaller scales in many parts of the world. In Australia, although monitoring is widely discussed, a lack of both long-term monitoring programs, poor execution of monitoring programs, and consequently, a paucity of consistently collected data from monitoring have undermined the effectiveness of government-led initiatives like State of the Environment Reporting (Lindenmayer et al., 2015), as well as billion dollar environmental restoration programs such as the Natural Heritage Trust (ANAO, 2008) and the Salinity Action Program (Pannell & Roberts, 2010). In the case of biodiversity conservation in Australia, comparatively low levels of conservation funding (Waldron et al., 2017) that is well below that needed to prevent extinctions (Wintle et al., 2019) is a major contributor to the lack of monitoring programs for threatened species. Indeed, the vast majority of Australia’s threatened vertebrates are not monitored well enough for managers to detect and respond to declines in their distribution and abundance (Scheele et al., 2019). A similar set of deficiencies besets Australia’s threatened plants (Lavery et al., 2021). The very limited number of monitoring programs for threatened species in Queensland is another prime example. The Queensland Audit Office (Queensland Audit Office, 2018) noted that robust monitoring data were available for only a few threatened species in Queensland and, with very few exceptions, the State Government “….does not know how threatened species are faring and whether management actions are having the desired impact” (p. 8). Queensland is not an isolated State in this context, as indicated by the recent, damming report on Victoria’s biodiversity strategy by the Victoria Auditor-General’s Office (Victoria Auditor-General’s Office, 2021) which stated that (the Victorian Government)… “cannot demonstrate if, or how well, it is halting further decline in Victoria’s threatened species populations” (p.1). In 2017, Australia lost its Long-term Ecological Research Network (Lindenmayer, 2017) in which key environmental management datasets were maintained to provide important insights into the condition of a wide range of ecosystems and threatened species across the continent (Lindenmayer et al., 2014).

Given the importance of monitoring for effective environmental management, periodically there are new government-led initiatives in Australia that seek to append major new monitoring programs to government investment in environmental management. Collectively, the authors of this article have witnessed several cycles of such initiatives over the past 40 + years, but these often fail to build on past lessons about what constitutes good monitoring and what should be avoided. Previously, we collectively have written about some of the features that should characterise successful monitoring programs, but poorly designed and implemented government-led programs continue to be implemented (e.g. Haywood et al., 2017), suggesting that past recommendations have failed to resonate with some policy makers and funding agencies. Here, in an attempt to improve monitoring practice in government-led monitoring initiatives (both in Australia and overseas), we outline how biodiversity monitoring programs can be best conceived, designed and implemented to maximize their chances of success. We do this by presenting eight things “never” to do in a biodiversity monitoring program. Our contribution is not intended to be an exhaustive treatment of the full array of factors that need to be considered as part of establishing and/or maintaining monitoring programs (entire books have been written on that topic; e.g. Gardner, 2010; Legge et al., 2018; Thompson et al., 1998). Rather, we provide a short précis of key recurring problems that have emerged in government-initiated monitoring programs. Our focus is on programmatic failures in government-level environmental monitoring programs in an Australian context, although we suggest that some of the general themes that characterise this article also will be relevant to other jurisdictions.

## Eight “nevers”

### #1. Never commence new environmental management programs without also committing to a monitoring program

A common failing of government-initiated conservation and environmental management programs is to consider monitoring design and data collection only after management is well underway. Part of the problem may lie with policy makers responding to political demands to quickly roll out large funding programs. Such haste can mean there is insufficient time taken to ensure that essential parts of those programs such as monitoring are well conceived and designed at the outset. There is also insufficient time taken for baseline data to be collected before management begins (if it does not already exist) so that the impact of the program can be determined. Australia’s multi-billion dollar Natural Heritage Trust program that, in part, aimed to promote widespread replanting efforts is but one of many examples (ANAO, 1997; Hajkowicz, 2009). Some limited monitoring started only several years after the program had commenced (Cunningham et al., 2007). Conversely, the Australian Government’s Environmental Stewardship Program, in which farmers were paid to implement management actions that had a positive effect on biodiversity on their land (such as livestock grazing control) (Burns et al., 2016), included a detailed monitoring program from its inception (Lindenmayer et al., 2012). This later monitoring program that was tied to management actions at the outset has produced some important insights into changes in vegetation condition and biodiversity response associated with management interventions (Kay et al., 2017; Sato et al., 2019). In particular, the design of the monitoring program with matched treatment and control sites was able to contrast the ongoing effects of business as usual livestock grazing on biodiversity with biodiversity in those in areas on the same farm where there was seasonally controlled grazing under stewardship protocols and which has led to evidence-based improvements in stewardship management of temperate woodlands (Lindenmayer et al., 2012, 2018a, b, c). Importantly, the use of monitoring was able to highlight differences in plant and bird responses under alternative grazing regimes during wetter periods and droughts.

We argue that for most new conservation initiatives and large, periodic environmental management programs, it is critical to set explicit measurable objectives that can then contextualize and inform the design of the monitoring that is required to evaluate the management program’s success or progress towards that success. Considering monitoring in initial program inception and design enables accurate costing of monitoring and ideally enables a “before investment” baseline to be measured. This improves the chances that monitoring will be properly designed, maximizing the chances of detecting a clear signal of management. A general rule of thumb is that approximately 10% of the total budget of management programs should be dedicated to monitoring the effectiveness of a program (Franklin et al., 1999), although this figure would need to be scaled relative to program budget and area covered by the program size.

#### Caveats

We acknowledge that in some cases, a monitoring program will need to be imposed on existing management that has been underway for a long time. An example is monitoring to quantify the impacts of forestry operations on populations of forest-dependent species, where the monitoring program must, to some extent, be retrofitted to address key questions. Under these circumstances, it is usually better to have late-imposed monitoring than no monitoring; statistical matching methods can be used to locate appropriate control sites (Schleicher et al., 2020), and in some instances trends in species or ecosystem attributes can be discerned even without baseline monitoring or “before impact” monitoring (e.g. Lindenmayer et al., 2020).

### #2. Never embark on a monitoring program without knowing what questions you are asking

This recommendation also could have been labelled “never collect a ‘mountain’ of data and then wonder what question to ask after those data have been gathered” which is, in essence, doing science backwards. This is a deep-seated problem, not only in Australia but elsewhere, with current trends away from monitoring programs that are well-designed and fit-for-purpose and towards Earth Observation Networks (such as within Terrestrial Ecosystem Research Network in Australia and NEON in the USA), where the fundamental aim is to generate, store, and share large amounts of data, especially using new kinds of sensor technologies, but without data collection being guided by well-crafted questions that would support improvements in programs (Lindenmayer et al., 2018a, b, c).

A good monitoring program needs to be clear about the question/s being asked as this will inform decisions about what data to collect, the experimental design to underpin data collection, and the likely cost of the program (Lindenmayer & Likens, 2018). For example, an important contrast between the Natural Heritage Trust and the Environmental Stewardship programs described above may have been the formulation of measurable objectives in the latter, but not the former. Where goals and objectives are poorly conceptualized, monitoring may be poorly anchored, limiting the ability to measure progress (McDonald-Madden et al., 2010).

A related problem is that in some cases, although questions are posed, they are framed too broadly or are imprecise (e.g. Are we conserving biodiversity?), and this precludes developing and then establishing monitoring programs that can effectively answer such questions. Good questions can be relatively simple, such as: Is a population at a particular site increasing? Is a particular management action such as weed control reducing the impact of weeds on species of interest? or Is a target species doing better at sites that are being managed compared to sites that are not? For example: Is placing more woody debris along creeks leading to an increase in the number of frogs compared to places where debris are not being added?

Monitoring should address questions that are management-relevant, that can improve management decisions and actions if answered, and that can provide rigorous evidence of the effect of management interventions. In a sobering example, Russell-Smith et al. (2003) lamented the fact that although a major, large-scale fire experiment in Australia’s Northern Territory produced interesting findings, they were of limited practical use because they did not relate well to the kinds of fire management typically applied in their study region. Conversely, there may be instances where a well-designed experiment may answer a given question far more efficiently than monitoring. A good example is the discovery of the ozone hole in Antarctica through a well-designed, question-driven experiment rather than detailed monitoring of many variables (Shanklin, 2010).

The key questions being addressed in a monitoring program may need to change over time, especially given the rate and magnitude of change currently being experienced in ecosystems worldwide, and the diversity and interaction of the factors driving change. In these cases, an adaptive monitoring approach (Lindenmayer & Likens, 2009) can be employed to help reshape monitoring programs to address new questions, without breaching the integrity of long-term datasets. In other cases, an existing monitoring program may need to be terminated and a new one commenced if more important new questions cannot be answered (but see “#5. Never change the way you monitor something part way through without ensuring new methods can be calibrated with the old ones”).

We acknowledge that some monitoring programs that have commenced without a question have led to other important discoveries. An example in an Australian context is a roadside mammal monitoring program in Tasmania designed to understand the impact of herbivore poisoning on native species, but resulted in the statistical corroboration of widespread declines in Tasmanian devil populations due to devil facial tumour disease (Hawkins et al., 2006; Wintle et al., 2010). However, monitoring programs underpinned by an explicit purpose will typically be far more effective than those without clearly articulated questions and a well-defined plan for addressing those questions with data (Lindenmayer & Likens, 2018; Nichols & Williams, 2006).

### #3. Never implement a monitoring program without designing it robustly to answer your questions

Some monitoring programs are poorly designed and unable to answer the key questions for which the monitoring program was proposed, even when those questions are sensible. As an example, the Victorian Forest Monitoring Program aims to “assess and monitor the extent, state and sustainable development of Victoria’s public forests in a timely and accurate manner”. The program comprises plots set across grid-based observational units throughout Victoria (Haywood et al., 2017). However, there are very few plots in areas where forestry operations are concentrated, such as the Central Highlands region of that State (where 65% of all logging in the State occurs). This has meant that the Victorian Forest Monitoring Program cannot answer fundamentally important questions such as those associated with the impacts of logging on biodiversity or provide biodiversity trends within key forest types where most timber harvesting takes place.

Poorly designed monitoring programs often have limited statistical power, precluding the ability to detect change (Southwell et al., 2019). They also commonly lack appropriate controls or appropriate contrasts (such as where a management action occurred and did not occur) and low levels of replication (if any), thereby limiting the ability to assign causality or to be confident that a key management intervention (like feral animal control or weed control) has been effective.

We argue that good experimental design should include consideration of how many sites need to be monitored, how much data need to be collected, and for how long monitoring needs to continue to be confident observed trends are real. The statistical analysis of whether or not a proposed monitoring program is likely to correctly describe trends and to infer the drivers of those trends, i.e. statistical power analysis, can now be performed using freely available software that can handle complex monitoring questions and designs (e.g. Southwell et al., 2019). Such power analyses were employed to assist in determining how well monitoring data would be able to detect declines in mammal populations in three national parks in the Northern Territory (Southwell et al., 2019).

Study design can be challenging as there are many factors to consider. These include (among others) the ability to actually answer the key questions being posed (see “#2. Never embark on a monitoring program without knowing what questions you are asking”), accounting for seasonal and/or annual variation relative to long-term trends, observer differences in measurement of flora and fauna (e.g. Cunningham et al., 1999; Gorrod & Keith, 2009), the detection of species relative to actual site occupancy (McKenzie et al., 2003), and the frequency of repeat surveys in a given sampling period. The best design will often be a BACI design, which involves monitoring at sites before and after a given management action was implemented and monitoring at “control” sites where there has been no management intervention. This design allows for the influence of the management program to be distilled from natural or background variation (Faith et al., 1991). In some circumstances, such as those monitoring programs that seek to quantify the landscape-scale effects of logging, control sites (where there is no logging) may need to be established in areas such as national parks and reserves. A key point here is that it is critical to ensure that monitoring occurs in many parts of the public land estate, including in national parks and reserves (where human land uses like logging are now excluded) but where there generally has been a poor record of robust, long-term monitoring to date (e.g. see: Queensland Audit Office, 2018). However, study design in these cases must account for potential confounding factors, for example, wood production forests tend to be in higher productivity areas relative to reserves (see Braithwaite, 1984). Hence, a lack of difference in fauna between tenures may be a function of productivity differences or of populations in wood production being reduced to similar levels typical of less productive national parks (Lindenmayer & Laurance, 2012).

We note that measurements at just two points in time are usually *not* adequate monitoring, even if those points are a long way apart. This is because there can be an almost infinite number of different trajectories for a line between two points (Stuble et al., 2021). We acknowledge that two samples can be better than one sample, but the value of studies with two samples can be influenced by whether a key objective of a monitoring program is to quantify, for example, the net change in a population or the shape of the temporal trajectory in that population (Stuble et al., 2021).

### #4. Never ignore the importance of matching the purpose and objectives of a monitoring program to the design of that program

New monitoring programs will almost always need to be designed and implemented for a given purpose. Rarely can monitoring programs come “straight off a shelf” of standard designs and delivery. This is because of complexities of study design, addressing well considered questions (of management relevance but which will often be context specific), the particular attributes or variables being targeted for measurement, and the method of measurement (e.g. a species, community, ecosystem, or threatening process). Therefore, because a monitoring program worked in one place or on a given species does not mean it will automatically work again under a different set of circumstances. Of course, this does not mean there are not valuable lessons to be learned from other programs—but rather that context is critical. Therefore, we argue there is a need to be wary of adopting ‘standard’, ‘generic’, or ‘multi-purpose’ monitoring protocols or any protocol applied elsewhere without critically evaluating whether they appropriate to achieve key objectives. For example, typical multi-species programs using randomly selected sites in space monitored across seasons are not optimal for many species. In the case of the threatened Brush-tailed Rabbit-rat *Conilurus penicillatus* on the Northern Territory’s Tiwi Islands, a far better option was a targeted monitoring program focussing on suitable *C. penicillatus* habitat in the late dry season when the species was more easily detected. This approach required half as many sites to detect the same level of population change (Geyle et al., 2018).

Some government-initiated monitoring programs attempt to employ generic approaches that are characterized by an ill-informed fascination with new and emerging technologies that are poorly suited to the entities that need to be monitored. A classic example was the Australian Government’s roll-out of Cybertracker technology to record geo-locational information under the Indigenous Protected Area, Working on Country and National Heritage Landscape programmes (Wilson, 2015). To the best of our collective knowledge, a decade after this deployment in technology, there appears to be very little insight of monitoring and management value to show from this investment.

Critical evaluation may reveal that some elements of a standard protocol might be fit for purpose, others not. An example of these kinds of issues is the PPbio monitoring protocol that was developed in tropical South America (Magnusson et al., 2008) and then adopted in south-eastern Queensland (with an accompanying field manual of protocols) (Hero et al., 2014). Whilst the notion of standardised monitoring protocols is attractive, in this (and other cases), the ways of surveying biota in one ecosystem (e.g. tropical rainforests in South America) were unsuitable in an entirely different system (such as temperate open forests in eastern Australia) with very different elements of biota, different underlying ecological processes, and different threats and drivers of change (Lindenmayer & Likens, 2018).

### #5. Never change the way you monitor something part way through without ensuring new methods can be calibrated with the old ones

The way data are collected can have an important influence on the interpretation of those data. Changing the methods used to gather data can breach the integrity of a time series and confound time with survey methodology. This can make it difficult to determine whether changes over time are real or simply an artefact of a change in the methods used. A highly controversial example of this problem concerns levels of silicon in Lake Michigan in the USA where major changes coincided with a change in the laboratory analysing the water samples (Shapiro & Swain, 1983). Despite ongoing court cases, establishing whether there have truly been environmental changes in silicon levels remained unresolved.

If existing monitoring programs or the measurement methods that underpin them are found wanting or inefficient in their capacity to answer key monitoring questions, design changes may be needed. This can be part of the Adaptive Monitoring approach that was described in “#2. Never embark on a monitoring program without knowing what questions you are asking” above. For example, a species may become increasingly rare over time, rendering the initial design insufficiently powerful, or a new method of detecting a species (e.g. eDNA or camera traps) may prove to be much more cost-effective and accurate. In such cases, a new method should be deployed only after a careful process of calibration to ensure that old and new data are fungible. Calibration involves a period of time in which both established and novel methods are used to limit the probability of confounding between the influence of time, management actions, and the methods of measurement. This can help maintain the integrity of long-term datasets. As an example, the Queensland Government’s Statewide Landcover And Trees Study (SLATS) is a long-term monitoring program that reports on annual woody vegetation cover change across the state, including attributing changes to land clearing. The program has transitioned from using Landsat satellite series to also using Sentinel-2 satellite imagery, captured every 5 days for the entire state. As the program tracks change over time, the Landsat data will continue to be used in addition to Sentinel-2, to provide ongoing compatibility with the existing time series.

Finally, maintenance of consistency in field methods or a change in field methods demands good (and readily accessible) descriptions of meta-data and field survey protocols, enabling work to be continued by others. This is especially critical when the management of a monitoring program is transitioned to a new leadership team.

### #6. Never try to monitor everything and avoid other kinds of over-commitments

Many monitoring programs make serious over-commitments at the outset and are unable to deliver on them, or they deliver on them poorly. For example, some monitoring programs try to monitor too many things or too many time-intensive measurements for a given entity (see the example below). This can lead to the sampling burden becoming unsustainable or result in insufficient data being collected for useful inference about anything. We argue it is better to monitor a smaller number of entities well than a large number poorly. Decisions about which entities make that subset should be guided by the questions being posed, available budget, statistical design, and practical realities associated with field sampling (e.g. quantifying species diversity in invertebrates can be almost taxonomically intractable, whereas it is far more straightforward for mammals and birds). Much of the uncertainty about how much monitoring could and should be achieved can be addressed using thoughtful consideration of the costs and effort required to complete various levels of sampling and a priori power analysis (Southwell et al., 2019).

Beyond the mistake of creating long lists of entities to be measured, other kinds of over-commitments can include excessive frequency of monitoring and inaccessibility of large numbers of sites. A well-intentioned monitoring program in Alberta, Canada provides an example of this. A large number of entities was targeted for monitoring (from mites, springtails, and fungi to vascular plants, birds, and mammals) at 1,656 sites, many remotely located across the entire province (Alberta Biodiversity Monitoring Institute, 2009). The monitoring program was unwieldly and unsustainable and has had to be reduced substantially (Lindenmayer & Likens, 2018).

Some of the kinds of over-commitment problems outlined above can be solved by using non-standard experimental designs. These include a rotating sample framework in which a subset of a population of monitoring sites is sampled in a given year (or other time period) as has been applied in the case of seabirds on islands which can be logistically and financially challenging to access (Welsh et al., 2000). These issues of over-commitment underscore the importance of ensuring that the demands of a monitoring protocol are guided by financial and logistical constraints so that the monitoring program can be adequately sustained over time.

The link between monitoring and management also highlights that it is often critical to monitor not only the responses of key components of biodiversity but also the threats to biodiversity, together with management actions that aim to mitigate the threats. This can allow preliminary evaluations of the likely effectiveness of management interventions, even if the ultimate biodiversity response is unlikely to be evident for many years or decades (Mayfield et al., 2020). A useful example is the effects of domestic livestock grazing on biodiversity in which it is important to monitor: (1) grazing intensity, (2) efforts to control grazing pressure (such as the establishment of fences) and the time elapsed since a management intervention occurred, (3) changes in vegetation cover linked to grazing, and (4) the direct effects (e.g. through trampling of bird nests) and indirect effects (e.g. mediated through changes in habitat attributes like leaf litter and midstorey vegetation) on biota (Lindenmayer et al., 2018a, b, c).

### #7. Never collect data without planning to curate the data, properly store it, and report on it

Some long-term monitoring programs gather large amounts of data that are never analyzed or made public. We argue that the use of taxpayer and/or private donor funding in monitoring programs comes with a moral (and often a contractual) responsibility to properly curate those data, and make them widely available, and ensure they are discoverable by the public and future land managers and researchers, including those who wish to maintain or re-establish a long-term study. Part of this responsibility lies with the funders themselves—to ensure that supporting the data curation and storage as well as analysis and public reporting of results is as essential as supporting the monitoring itself. A centralized, national facility to collect, curate, analyse, and provide access to biodiversity data collected during monitoring attached to major government investments in environmental management would ensure that information is not lost between the end of one program and the beginning of the next. Such a facility could support many functions relating to environmental management (Australian Academy of Science, 2020; Binskin et al., 2020).

Allied with this is a need to describe those data and associated field protocols, so that others can continue the data collection process and/or re-analyse those data at a later stage, and a comparable design can be employed by others working on similar questions elsewhere. Effective long-term storage, detailed meta-data, and curation of data are likewise important, to ensure that it is discoverable by future researchers, including those who wish to maintain or re-establish a long-term study. Data curation can be time-consuming and costly, and it is important the budget for monitoring programs makes allowance for these key tasks; it may be up to 20% of total funding allocated to monitoring (Berman & Cerf, 2013).

### #8. Never start a monitoring program without understanding how you will resource it

We believe that monitoring programs should be an integral component of all environmental management initiatives and allocated sufficient resources to meet their objectives. Well-designed monitoring can deliver a strong return on investment in environmental and biodiversity management—but adequate resourcing is needed to ensure the quality of monitoring is sufficient for results to be useful. Where the needed resources are not provided, monitoring will typically fail to achieve program-level objectives, with such failure contributing to a reputation that monitoring is a waste of money. The available budget can be scaled to the monitoring needs.

Biodiversity change may be multi-decadal, but most biodiversity management and monitoring programs are resourced over much shorter periods (typically 1 to 3 years), creating a mismatch between objectives, outcomes, and resourcing. As stated above, monitoring is rarely properly funded, usually the last item considered in budgets, and the first thing cut when there is a budget problem (Lindenmayer & Likens, 2018). Monitoring does not have to be expensive to be effective. For example, the national Malleefowl adaptive management experiment in Australia is largely monitored by volunteer groups using visual observation that requires no expensive equipment (Hauser et al., 2019). However, in cases where much of the monitoring will be conducted by volunteer citizen scientists, there is a need to be aware of potentially large differences in data quality among different observers and to determine whether such differences will have a major impact on outcomes. In other cases, citizen science data may be unsuitable for use in some key tasks linked to monitoring programs like determining population trends over time. This proved to be the case for a large proportion of the datasets that have been gathered on birds in Australia as it remains unclear if the same sites had been monitored repeatedly over time, if field methods were consistent, and if a standard taxonomic classification had been applied across all records (Bayraktarov et al., 2019).

Finally, we suggest that where there are plans to defund an existing monitoring program, the consequences of doing so need to be made clear to those responsible for budget allocations. In cases where there is a risk of major reductions in funding, innovative designs could be considered, such as rotating sub-sampling to enable a program to persist without compromising the creation of robust time series data, albeit with a smaller budget (e.g. Lindenmayer et al., 2012). There also can be value in developing a strategy for project “mothballing” so that a program can be resuscitated when funds once again become available. Such mothballing activities include documentation of where sites are located, how data collection is conducted, and how and where data are stored.

## Discussion

Biodiversity is declining at an accelerating rate, and there is increasing need for more effective programs to staunch this loss (Intergovernmental Science-policy Platform on Biodiversity and Ecosystem Services (IPBES), 2019; Maxwell et al., 2016). Effective environmental management, threatened species recovery, and ongoing and increased funding all depend on good monitoring that measures the performance of conservation investments (e.g. Gardner, 2010; Garnett et al., 2018; Legge et al., 2018; Spellerberg, 1994). Monitoring has sometimes been called the “Cinderella Science” (Nisbet, 2007). However, we argue that this is a misnomer in many ways. It is not neglected because there is a vast literature on the importance of monitoring; numerous strategic documents discuss it (e.g. the Convention on Biological Diversity) and many programs have commenced. Moreover, there is no “handsome prince on the horizon” and good monitoring will not live happily ever after without constant vigilance, re-appraisal, affirmation of its value, and relevance to environmental management.

At a national level, Australia’s lack of attention towards robust biodiversity monitoring programs has undermined the reportability, and even the success, of many of the nation’s environmental management programs (including billion dollar ones) such as the Natural Heritage Trust, Caring For Our Country, The Biodiversity Fund, the National Landcare Program, and threatened species recovery programs (ANAO, 2008; Scheele et al., 2019). Inadequate data collection and analysis continues to undermine State of Environment Reports (Lindenmayer et al., 2015) and a massive shortfall in funding means that many threatened species are poorly monitored in Australia (Scheele et al., 2019; Wintle et al., 2019). These problems also characterize not only National initiatives, but State-level environmental programs including those associated with threatened species management (e.g. see: Queensland Audit Office, 2018; Victoria Auditor-General’s Office, 2021). Recently proposed industry initiatives such as sustainability frameworks in the beef and sheep industries in Australia (see https://www.sustainableaustralianbeef.com.au/) will likely fail because there is a paucity of well-designed environmental monitoring throughout the large areas of the Australian continent subject to livestock grazing (Williams & Price, 2011). Similarly, there are plans to develop environmental prediction systems in Australia, but these too will have limited accuracy and value in the absence of well-designed environmental monitoring to provide the long-term data needed to develop such systems (Lindenmayer, 2018).

We recognize there is no such thing as a perfect monitoring program, but we suggest that at least considering the eight “nevers” in this article and their solutions (Table 1) will help build on the experiences of past monitoring programs and studies to improve future ones. Until proper attention is paid to these key issues and good practice, institutionalized, large-scale environmental management programs will often continue to repeat past mistakes. Moreover, poorly implemented monitoring will typically not provide answers to important questions and will squander effort, resources, and goodwill.

**Table 1:** A counter to the eight “nevers”: key components of effective environmental monitoring programs and strategies to avoid succumbing to the “nevers”

1. At the outset of an environmental management program, make monitoring part of the program design - Dedicate approximately 10% of the total budget for a management program to monitoring management effectiveness (may need to be scaled relative to program size, budget, and/or area covered) - Define goals and objectives for the environmental management program to ensure monitoring is well anchored and progress can be measured towards quantitative goals (e.g. the restoration of populations of a target species or a given size) - If necessary, retrofit late-imposed monitoring as this is always better than no monitoring
2. Be explicit about the purpose and capability of the monitoring program - Define some management-relevant questions at the outset, aim to assess interventions, and improve decisions and actions - Employ an adaptive monitoring approach to help reshape monitoring programs as new questions arise, without breaching the integrity of long-term datasets
3. Ensure there is a robust experimental design underpinning the monitoring program - Consider study design carefully so that key questions can be answered with the data. Consider the range of important variables including seasonal and/or annual variation, observer differences, detectability vs site occupancy of species, and number of sites and frequency of sampling required - A BACI design is often ideal, involving monitoring before and after a given management action with “control” and “treatment” sites - Two sampling points in time are better than one, but aim for more to detect both net changes and the temporal trajectories of change
4. Ensure the purpose and objectives of a monitoring program match the design of that program - Borrow from successful examples of monitoring where possible, but ensure the specific biota, underlying ecological processes, and threats and drivers of change are all carefully considered and used as the framework around which monitoring is designed
5. Maintain the integrity of data collection and curation - For any change, calibrate by incorporating a period of time in which both established and novel methods are used simultaneously - Maintain a good and readily accessible description of meta-data and field survey protocols
6. Focus on a subset of key entities to measure (and measure them well) - Guide the selection of monitoring entities by identifying key components of biodiversity, threats, and the management actions that will aim to mitigate threats - Solve over-commitment problems using non-standard experimental designs such as using a rotating sample framework (e.g. a subset of monitoring sites in any given year)
7. Plan to analyze data from a monitoring program and publicly report - From the outset ensure accurate meta-data are associated with the program, describe in detail the monitoring data being collected and the associated field protocols to increase utility of the data and program - Allocate up to 20% of total program funding to long-term storage, detailed meta-data, and curation of data. This may include new infrastructure for enduring data access and reporting
8. Determine the budget required for an adequate monitoring program that is fit-for-purpose, and ensure that budget is secured - Innovative designs can be considered (e.g. rotating sub-sampling) where there is a risk of major funding reductions - “Mothballing” may be one strategy to temporarily preserve a program under funding cuts, ready to restart once funding becomes available again

We argue that the culture leading to poor quality monitoring in Australia (and many other parts of the world) needs to change in line with public expectations so that the benefits of conservation programs can be demonstrable, auditable, and rigorously reported and that the causes of failure can be discerned and corrected (ANAO, 2008; Hajkowicz, 2009; Pannell & Roberts, 2010; Queensland Audit Office, 2018). In areas such as threatened species management, robust monitoring programs should be a mandatory part of recovery efforts and legislated under environmental laws, as occurs in the USA (Wintle, 2018; Wintle et al., 2019). Similarly, well-designed and rigorously implemented, and maintained monitoring programs must be part of all new government-led initiatives in environmental management at state and national levels in Australia. Well-designed government-initiated environmental monitoring also must be part of public land management, including national parks and nature reserves, especially given the stresses acting upon many natural areas in Australia ranging from climate change to altered fire regimes and the impacts of invasive species, together with the compounding effects of all of these drivers (Bergstrom et al., 2021).

Part of the solution to the perennial problem of limited monitoring or poorly designed monitoring is the development of stronger scientist-policy maker and scientist-manager partnerships. This is essential to ensure that scientific and statistical principles of design and practice can be injected into new proposals to instigate new large-scale government-led monitoring programs and efforts to renovate existing programs to increase their effectiveness and robustness. This is a call for some scientists to dispense with the notion that monitoring is second rate science. There is also a role for policy makers and resource managers (as well as scientists) to look more broadly than their regions, states, or nationally and discern what monitoring programs have worked and not worked where and “steal” the good innovations that are effective (e.g. such as in Switzerland (Federal Office for the Environment, 2017)). This includes actually acting on, not ignoring, the findings of independent assessments such as those by audit reports (Queensland Audit Office, 2018; Victoria Auditor-General's Office, 2021). Indeed, we suggest there is an important and increasingly greater role for auditing in environmental management initiatives to expose those government programs that lack monitoring or where monitoring efforts are poor and ineffective (e.g. ANAO, 1997, 2008; Queensland Audit Office, 2018; Victoria Auditor-General's Office, 2021). Critically, there needs to be mechanisms developed to force governments to change the way they implement new environmental initiatives and rejuvenate existing failing ones to account for the findings from audit reports. Sadly, the scathing findings by the (Victoria Auditor-General's Office, 2021) are little different to previous reports on the poor quality of environmental programs and monitoring in that State, indicating limited progress or improvement over the past decade.

The incentive to do monitoring in government-led environmental programs might be increased if monitoring data can be used in other ways that highlight their value. As an example, there has been increasing interest in environmental and economic accounting (Vardon et al., 2021) in which the various values of natural assets (including biodiversity) are made explicit in an accounting context for policy makers and resource managers (see Keith et al., 2017)). Building such kinds of accounts depends on data from well-designed monitoring programs and emphasizes the importance of the latter.

Another solution to the ongoing problems of such a poor record on monitoring could be to consider entirely new institutional models, like that for the Australian Bureau of Statistics (which is an independent statutory body). The Bureau of Meteorology has successfully prosecuted the case for supporting the collection and analysis of long-term climate and weather data. An equivalent agency responsible for environmental and conservation monitoring programs would be a major positive step towards rectifying past problems. In fact, the Australian Academy of Science has recommended a new institution akin to the Bureau of Meteorology to support monitoring of new government-led environmental management initiatives (Australian Academy of Science, 2020). This kind of institution could provide a strong template for how a public environmental data and management effectiveness body could help make monitoring meaningful in Australia.

## Conclusions

Poorly designed and/or implemented monitoring programs have characterized many large-scale environmental management initiatives around the world. Based on our collective experiences in an Australian context, we outline eight things that should never be done in an environmental monitoring program if the aim is for it to be useful. Failure to address these key issues will mean that institutionalized, large-scale environmental management programs will often continue to repeat past mistakes and will squander effort, resources, and goodwill. We balance our “nevers” with a checklist of actions (see Table 1) that should increase the chances a monitoring program will measure the effectiveness of environmental management. We believe that scientists and resource management practitioners need to more stridently advocate for and play a more active part in well-designed, implemented, and maintained monitoring programs.
